# Predictors of the Intention to Stop Using Smart Devices at Bedtime Among University Students in Saudi Arabia: Cross-Sectional Survey

**DOI:** 10.2196/67223

**Published:** 2025-03-10

**Authors:** Manal Almalki

**Affiliations:** 1 Public Health Department College of Nursing and Health Sciences Jazan University Jazan Saudi Arabia

**Keywords:** smart devices, smartphone, digital health, digital technology, sleep quality, university student, bedtime habits, Saudi Arabia, path analysis, sleep disturbances, well-being, usage, intention, behavior, mobile phone

## Abstract

**Background:**

The widespread use of smart devices, particularly among university students, has raised concerns about their impact on sleep quality. Bedtime usage of smart devices is associated with sleep disruptions and poor sleep quality.

**Objective:**

This study aimed to explore the behavioral and perceptual factors influencing university students’ intention to stop using smart devices at bedtime in Saudi Arabia.

**Methods:**

A cross-sectional survey was conducted in June 2024 and distributed via social media platforms to university students (aged ≥18 years). The questionnaire collected data on demographics, smart device usage habits, perceived negative effects on sleep, and physical sleep disturbances. The Pittsburgh Sleep Quality Index was used to assess sleep quality. Path analysis was performed to evaluate relationships between the outcome variables, intended to stop using smart device usage, and 3 latent variables: sleep quality smartphone usage, sleep quality perceived negative effects, and sleep quality during the past month. Model fit was assessed using chi-square, comparative fit index, and root mean square error of approximation.

**Results:**

Of the 774 participants, 90.43% (700/774) reported using their smart devices every night and 72.48% (561/774) believed bedtime device use negatively affected them the next morning. The most frequently reported next-morning symptoms were fatigue or drowsiness (480/774, 62.01%). Common purposes for bedtime device use were staying in touch with friends or family (432/774, 55.81%), entertainment (355/774, 45.86%), and filling up spare time (345/774, 44.57%). Overall, 58.26% (451/774) expressed an intention to stop bedtime device use within the next 3 months. Path analysis demonstrated that frequent nightly use (path coefficient=0.36) and after-lights-off usage (0.49) were positively associated with the intention to stop, whereas spending ≥3 hours on devices (–0.35) and engaging in multiple activities (–0.18) had negative associations. The strongest predictors of the intention to stop were perceived negative effects on next-morning well-being (0.71) and difficulty breathing comfortably during sleep (0.64). Model fit was excellent (comparative fit index=0.845 and root mean square error of approximation=0.039).

**Conclusions:**

Perceived negative effects on sleep quality and physical sleep disturbances are strong predictors of the intention to stop using smart devices at bedtime among university students in Saudi Arabia. Interventions aimed at improving sleep hygiene should focus on raising awareness about the impact of smart device use on well-being and addressing behaviors such as late-night usage and heavy screen time. Public health strategies should target both psychological and physiological aspects of bedtime smart device use to improve sleep quality in this population.

## Introduction

In the digital age, smart devices such as smartphones, tablets, and laptops have become integral parts of daily life, especially among university students [1]. These devices offer constant connectivity, enabling students to engage in academic activities, communicate with peers, and access entertainment. In Saudi Arabia, where more than 97% of the population uses smartphones [2], smart devices have become an essential tool for both learning and social interaction. On a global scale, the number of smartphone mobile network subscriptions exceeded 7 billion in 2023 and is anticipated to exceed 7.7 billion by 2028 [3].

University students, often facing academic pressures and irregular schedules, are particularly vulnerable to the excessive use of smart devices at bedtime [4-6]. They may engage in multiple bedtime activities, such as social media browsing, texting, gaming, or even frequent awakenings to check notifications, which counteract the body’s natural inclination to relax and fall asleep [7-9]. These behaviors are further intensified by the immediate gratification of staying connected and creating a cycle that sustains poor sleep practices [8-10]. In Saudi Arabia, smart devices often serve as a primary means of students’ communication, relaxation, and staying in touch with friends and family [11], which further reinforces their use, even during late-night hours [12].

The excessive use of these smart devices, particularly during bedtime, raises concerns about their impact on students’ sleep quality [4], overall well-being [5], and academic performance [6]. Late-night screen exposure can disrupt the body’s circadian rhythm through both blue light emissions and heightened mental stimulation [13], often delaying sleep onset and reducing total sleep duration [14-17]. Over time, these disruptions can lead to chronic sleep debt, making students more prone to daytime drowsiness, poor concentration [4,5], and lower academic achievement [14,15]. Moreover, poor sleep quality can contribute to the development of mental health issues such as anxiety and depression [5], while these conditions can further disrupt sleep patterns [16]. This mental stimulation can lead to increased cognitive arousal and addiction, which is detrimental to initiating and maintaining sleep [7,8,17]. This addiction does not only affect the quantity of sleep but also its quality, leading to a less restorative sleep experience [1,4,18].

Despite growing awareness of the potential harms of excessive screen time, many students continue to use smart devices at bedtime [13]. Understanding the factors that drive students to continue or stop using these devices at bedtime is crucial for addressing the sleep-related issues prevalent in this population [19,20]. According to the Theory of Planned Behavior [21], individuals’ intentions to engage in or discontinue a behavior are shaped by their attitudes toward the behavior, perceived control over it, and social norms. In the context of smart device use, it is likely that students’ perceptions of how device use affects their sleep and well-being, as well as their ingrained habits, play a key role in their decision to reduce or stop using smart devices at bedtime.

This study aims to examine the factors that predict the intention to stop using smart devices at bedtime among university students in Saudi Arabia. Specifically, it investigates the relationship between 3 key domains: (1) smart device usage behaviors (eg, frequency, duration, and activities performed on devices), (2) perceived negative effects of smart device use on sleep quality and next-morning well-being, and (3) physical sleep disturbances experienced during the past month. Through path analysis, this study seeks to understand the complex interplay between these variables and provide insights that can inform public health interventions targeted at improving sleep hygiene among Saudi university students.

## Methods

### Study Design

In this study, a cross-sectional survey of university students was conducted in June 2024 in Saudi Arabia. The STROBE (Strengthening the Reporting of Observational Studies in Epidemiology) guidelines of cross-sectional studies were followed to report our study’s findings [22]. The questionnaire was piloted with a small group of university students (n=7) to ensure clarity and relevance, with feedback used to refine its wording and structure.

### Study Recruitment

Participants were recruited using convenience sampling. A link to the questionnaire was posted on social media platforms such as WhatsApp (Meta), Snapchat, Telegram, and X (formerly known as Twitter). These platforms were chosen due to their popularity among university students in Saudi Arabia [23,24]. The link was accompanied by a recruitment message that outlined the study’s objectives, eligibility criteria, and an assurance of confidentiality. Eligible participants were students aged 18 years or older, currently enrolled in universities in Saudi Arabia, and capable of providing informed consent before starting the survey.

### Ethical Considerations

Ethical approval for the study was obtained from the Standing Committee for Publication and Research Ethics at Jazan University (reference number REC-45/11/1143). On the opening page of the survey, participants were presented with information about the study’s objectives and scope, followed by an informed consent statement. Participants were free to withdraw at any point without consequence. Also, participation was anonymous and no personally identifiable information was collected or used. No monetary or nonmonetary incentives were offered. Finally, this paper does not include any images or material in which participants can be identified.

### Questionnaire Design

The questionnaire was created using a Google Form, available in both English and Arabic, and it consisted of 4 sections. The first section gathered demographic data such as gender, age, marital status, highest level of education, residential region, health status, and employment status.

The second section examined participants’ smart device usage during bedtime during the past month. Questions covered the type of smart device most frequently used, the average frequency of use per week, time spent on the device at bedtime (ie, categorized as “less than 2 hours” or “3 hours or more”), usage after lights were turned off, the use of headphones, and other related behaviors. This section also addressed the activities participants engaged in while using their devices (eg, chatting on messaging platforms, watching videos, and setting alarms or reminders), as well as the purposes of smart device use before sleep (eg, education, entertainment, business, or work networking).

The third section explored participants’ perceptions of how smart device usage at bedtime affected their sleep quality, sleep duration, and overall health. The final section used the Pittsburgh Sleep Quality Index (PSQI) to assess participants’ physical sleep disturbances experienced during the past month. The PSQI consists of 19 self-rated items that evaluate subjective sleep quality, sleep latency, habitual sleep efficiency, sleep disturbances, use of sleep medications, and daytime dysfunction [25]. The Arabic-translated version of the PSQI, which has been validated for use in research, was used in this study [26].

### Statistical Analysis

Incomplete surveys were excluded. A power calculation using G*Power (α=.05, power=80%, moderate effect size, odds ratio [OR 1.5]) indicated a minimum sample size of 384 participants. The final sample size was 774, which exceeded this requirement, ensuring sufficient power to detect significant associations.

Categorical variables were summarized as frequencies and percentages. ORs with 95% CI values were calculated to assess the relations and their strengths between variables. A path analysis was then conducted to understand the complex relationships between the measured variable intended to stop using smart device usage, latent variables sleep quality smartphone usage (SQSU), sleep quality perceived negative effects (SQPNE), and sleep quality during the past month (SQDPM). This method was chosen because it allows for the simultaneous analysis of multiple dependent and independent variables, providing a comprehensive understanding of direct and indirect relationships between variables.

A typical path modal sequencing equation modeling (SEM) plot is constructed by defining a multiple set of regression formulas. Model fit was assessed by chi-square, comparative fit index (CFI), and root mean square error of approximation (RMSEA). The value of RMSEA less than 0.05 with CFI equal to 0.845 or higher with nonsignificant chi-square statistic indicates a good model fit. Data were coded, validated, and analyzed using IBM SPSS software, version 27.0 for Windows (IBM Corp). Plots were constructed using R software (version 4.1.2; R Core Team) with Lavaan and Semplot R packages. Two-tailed *P* value <.05 was considered to denote statistical significance.

## Results

### Participant Characteristics

A total of 774 participants completed the questionnaire, as shown in Table 1. Over half of them (451/774, 58.26%) expressed an intention to stop using smart devices during bedtime within the next 3 months. None of the demographic characteristics was associated with the acceptability of stopping the use of smart devices during bedtime; therefore, OR analysis is removed from Table 1.

The majority (397/774, 88.03%) were aged 24 years or younger, 74.72% (337/774) were female, and 86.92% (392/774) were unmarried. More than three-quarters of participants (657/774, 84.88%) had an undergraduate degree or higher, and 87.62% (283/323) did not intend to stop using smart devices at bedtime. Likewise, more than three-quarters (688/774, 88.89%) of participants who did not have any sleep disorders had 38% higher odds of intending to stop using smart devices at bedtime compared to participants with medically diagnosed sleep disorders. The most common sleep duration among all participants was 5-6 hours, with 34.88% (270/774) reporting this sleep range. Participants who intended to stop using smart devices at bedtime had a slightly higher percentage (176/451, 39.02%) of reporting 5-6 hours of sleep compared to those who did not intend to stop (94/323, 29.10%).

**Table 1:** Sample demographic characteristics of university students in Saudi Arabia.

Variables			Values (N=774)		Likely to stop using smart devices during bedtime in the next 3 months		
					Yes (n=451)		No (n=323)
**Age (years), n (%)**							
	18-24	681 (87.98)		397 (88.03)		284 (87.93)	
	≥25	93 (12.02)		54 (11.97)		39 (12.07)	
**Sex, n (%)**							
	Male	190 (24.55)		114 (25.28)		76 (23.53)	
	Female	584 (75.45)		337 (74.72)		247 (76.47)	
**Marital status, n (%)**							
	Married	91 (11.76)		59 (13.08)		32 (9.91)	
	Not married	683 (88.24)		392 (86.92)		291 (90.09)	
**Highest level of education, n (%)**							
	Diploma after high school or below	117 (15.12)		77 (17.07)		40 (12.38)	
	Undergraduate studies or higher	657 (84.88)		374 (82.93)		283 (87.62)	
**Residential region, n (%)**							
	North	47 (6.07)		30 (6.65)		17 (5.26)	
	South	382 (49.35)		223 (49.45)		159 (49.23)	
	East	21 (2.71)		13 (2.88)		8 (2.48)	
	West	69 (8.91)		39 (8.65)		30 (9.29)	
	Middle	255 (32.94)		146 (32.37)		109 (33.75)	
**Medically diagnosed with any sleep disorders, n (%)**							
	Yes	86 (11.11)		56 (12.42)		30 (9.29)	
	No	688 (88.89)		395 (87.58)		293 (90.71)	
**Sleep hours per day or night, n (%)**							
	Less than 5 h	118 (15.25)		68 (15.08)		50 (15.48)	
	5-6 h	270 (34.88)		176 (39.02)		94 (29.10)	
	7-8 h	245 (31.65)		138 (30.60)		107 (33.13)	
	9-10 h	74 (9.56)		40 (8.87)		34 (10.53)	
	More than 10 h	67 (8.66)		29 (6.43)		38 (11.76)	
**Employment status, n (%)**							
	Employed	210 (27.13)		131 (29.05)		79 (24.46)	
	Not employed	564 (72.86)		320 (70.95)		244 (75.54)	
**Job has night shifts (n=210), n (%)**							
	Yes	51 (24.28)		32 (24.43)		19 (24.05)	
	No	159 (75.71)		99 (75.57)		60 (75.95)	

### Smart Device Usage Behaviors

More than a ninth decile of participants (737/774, 94.83%) used a smartphone, followed by 3.74% (29/774) using a tablet, and only 1.03% (8/774) using a laptop, as shown in Table 2. The groups who were less willing to stop using smart devices during their bedtime in the next 3 months, in descending order, were participants who used their devices 1-3 nights per week (OR 0.05, 95% CI 0.00-0.43; *P<*.001), participants who used their devices 4-6 nights per week (OR 0.48, 95% CI 0.25-0.90; *P*=.02), participants who did not frequently use devices after lights were turned off (OR 0.52, 95% CI 0.33-0.83; *P<.*001), participants who did not often use headphones during bedtime (OR 0.73, 95% CI 0.55-0.97; *P=.*03), and participants who were not frequently awakened by smart devices at night (OR 0.71, 95% CI 0.53-0.95; *P=.*02). These groups showed significantly lower odds of intending to stop using their smart devices during bedtime compared to the respective reference groups.

**Table 2:** Smart device usage behaviors among university students in Saudi Arabia.

Variables					Values (N=774)		Likely to stop using smart devices during bedtime in the next 3 months						
							Yes (n=451)		No (n=323)		Odds ratio (95% CI)		*P* value
**Smart device type, n (%)**													
	Smartphone			737 (94.83)		434 (96.23)		303 (93.81)		Ref		—^a^	
	Laptop			8 (1.03)		2 (0.44)		6 (1.86)		4.29 (0.86-21.43)		.71	
	Tablet			29 (3.74)		15 (3.33)		14 (4.33)		1.33 (0.63-2.81)		.44	
**Frequency of using the smart device per week (SQSU^b^_1), n (%)**													
	Every night			700 (90.43)		392 (86.92)		308 (95.36)		Ref		—	
	4-6 nights			51 (6.58)		37 (8.20)		14 (4.33)		0.48 (0.25-0.90)		.02	
	1-3 nights			23 (2.97)		22 (4.88)		1 (0.31)		0.05 (0.00-0.43)		<.001	
**Time spent on a smart device at bedtime (SQSU_2), n (%)**													
	<2 h or less			494 (63.82)		309 (68.51)		185 (57.28)		Ref		—	
	3 h or more			280 (36.17)		142 (31.49)		138 (42.72)		1.62 (1.20-2.18)		<.001	
**Often using the smart device after lights are turned off (SQSU_3), n (%)**													
	Yes			674 (87.08)		380 (84.26)		294 (91.02)		Ref		—	
	No			100 (12.91)		71 (15.74)		29 (8.98)		0.52 (0.33-0.83)		<.001	
**Often using headphones during bedtime (SQSU_4), n (%)**													
	Yes			370 (47.80)		201 (44.57)		169 (52.32)		Ref		—	
	No			404 (52.19)		250 (55.43)		154 (47.68)		0.73 (0.55-0.97)		.03	
**Often awakened by the smart device at night (SQSU_5), n (%)**													
	Yes			309 (39.92)		165 (36.59)		144 (44.58)		Ref		—	
	No			465 (60.07)		286 (63.41)		179 (55.42)		0.71 (0.53-0.95)		.02	
**The most common posture when using the smart device, n (%)**													
	Lying down			668 (86.30)		383 (84.92)		285 (88.24)		Ref		—	
	Sitting			106 (13.69)		68 (15.08)		38 (11.76)		0.75 (0.49-1.14)		.20	
**Keeping the smart device while sleeping, n (%)**													
	In the bed			414 (53.48)		235 (52.11)		179 (55.42)		Ref		—	
	On the bedside table			316 (40.82)		186 (41.24)		130 (40.25)		0.91 (0.68-1.23)		.59	
	Far from the bed but inside the bedroom			44 (5.68)		30 (6.65)		14 (4.33)		0.61 (0.31-1.1)		.15	
**Most common smart device mode, n (%)**													
	Not general			586 (75.71)		336 (74.50)		250 (77.40)		Ref		—	
	General or vibratory			188 (24.28)		115 (25.50)		73 (22.60)		0.85 (0.60-1.19)		.39	
**Activities performed when using smart device during bedtime (SQSU_6), n (%)**													
	**(1) Setting alarms, reminders, and tasks**												
		False		309 (39.92)		173 (38.36)		136 (42.11)		Ref		—	
		True		465 (60.07)		278 (61.64)		187 (57.89)		0.85 (0.63-1.14)		.29	
	**(2) Chatting via WhatsApp, Snapchat, Telegram, etc**												
		False		269 (34.75)		170 (37.69)		99 (30.65)		Ref		—	
		True		505 (65.24)		281 (62.31)		224 (69.35)		1.36 (1.01-1.85)		.04	
	**(3) Debating and discussion via Twitter, WhatsApp**												
		False		594 (76.74)		346 (76.72)		248 (76.78)		Ref		—	
		True		180 (23.25)		105 (23.28)		75 (23.22)		0.99 (0.71-1.39)		1	
	**(4) Watching YouTube, TikTok, Instagram, etc**												
		False		276 (35.65)		178 (39.47)		98 (30.34)		Ref		—	
		True		498 (64.34)		273 (60.53)		225 (69.66)		1.49 (1.10-2.02)		<.001	
	**(5) Watching TV shows and movies**												
		False		470 (60.72)		298 (66.08)		172 (53.25)		Ref		—	
		True		304 (39.27)		153 (33.92)		151 (46.75)		1.70 (1.27-2.29)		<.001	
	**(6) Listening to the Qur’an and religious supplications**												
			False	606 (78.29)		349 (77.38)		257 (79.57)		Ref		—	
			True	168 (21.70)		102 (22.62)		66 (20.43)		0.87 (0.61-1.24)		.48	
	**(7) Listening to a podcast**												
			False	709 (91.60)		411 (91.13)		298 (92.26)		Ref		—	
			True	65 (8.39)		40 (8.87)		25 (7.74)		0.86 (0.51-1.45)		.60	
	**(8) Listening to music**												
			False	674 (87.08)		404 (89.58)		270 (83.59)		Ref		—	
			True	100 (12.91)		47 (10.42)		53 (16.41)		1.68 (1.10-2.57)		.01	
	**(9) Researching brands**												
			False	701 (90.56)		412 (91.35)		289 (89.47)		Ref		—	
			True	73 (9.43)		39 (8.65)		34 (10.53)		1.24 (0.76-2.01)		.38	
	**(10) Playing games**												
			False	657 (84.88)		391 (86.70)		266 (82.35)		Ref		—	
			True	117 (15.11)		60 (13.30)		57 (17.65)		1.39 (0.94-2.07)		.10	
	**(11) Surfing the internet**												
			False	604 (78.03)		359 (79.60)		245 (75.85)		Ref		—	
			True	170 (21.96)		92 (20.40)		78 (24.15)		1.24 (0.88-1.74)		.21	
	**(12) Browsing emails and text messages**												
			False	626 (80.87)		368 (81.60)		258 (79.88)		Ref		—	
			True	148 (19.12)		83 (18.40)		65 (20.12)		1.17 (0.77-1.60)		.57	
**Purposes** **of using the smart device during bedtime (** **SQSU_7** **)** **, n (%)**													
	**(1) Education and study**												
			False	453 (58.52)		258 (57.21)		195 (60.37)		Ref		—	
			True	321 (41.47)		193 (42.79)		128 (39.63)		0.87 (0.65-1.17)		.41	
	**(2) Staying in touch with friends and family**												
			False	342 (44.18)		204 (45.23)		138 (42.72)		Ref		—	
			True	432 (55.81)		247 (54.77)		185 (57.28)		1.10 (0.83-1.47)		.50	
	**(3) Following news and current**												
			False	498 (64.34)		292 (64.75)		206 (63.78)		Ref		—	
			True	276 (35.65)		159 (35.25)		117 (36.22)		1.04 (0.77-1.40)		.81	
	**(4) Sharing opinions and thoughts on social networks**												
			False	581 (75.06)		344 (76.27)		237 (73.37)		Ref		—	
			True	193 (24.93)		107 (23.73)		86 (26.63)		1.16 (0.83-1.62)		.39	
	**(5) Business or work networking**												
			False	680 (87.85)		390 (86.47)		290 (89.78)		Ref		—	
			True	94 (12.14)		61 (13.53)		33 (10.22)		0.72 (0.46-1.14)		.18	
	**(6) Researching how to do things**												
			False	660 (85.27)		386 (85.59)		274 (84.83)		Ref		—	
			True	114 (14.72)		65 (14.41)		49 (15.17)		1.06 (0.71-1.58)		.83	
	**(7) Shopping**												
			False	615 (79.45)		359 (79.60)		256 (79.26)		Ref		—	
			True	159 (20.54)		92 (20.40)		67 (20.74)		1.02 (0.71-1.45)		.92	
	**(8) Gaming**												
			False	619 (79.97)		374 (82.93)		245 (75.85)		Ref		—	
			True	155 (20.02)		77 (17.07)		78 (24.15)		1.54 (1.08-2.20)		.01	
	**(9) Entertainment**												
			False	419 (54.13)		275 (60.98)		144 (44.58)		Ref		—	
			True	355 (45.86)		176 (39.02)		179 (55.42)		1.94 (1.45-2.59)		<.001	
	**(10) Finding information**												
			False	603 (77.90)		365 (80.93)		238 (73.68)		Ref		—	
			True	171 (22.09)		86 (19.07)		85 (26.32)		1.51 (1.07-2.13)		.01	
	**(11) Being relaxed and calming down**												
			False	569 (73.51)		337 (74.72)		232 (71.83)		Ref		—	
			True	205 (26.48)		114 (25.28)		91 (28.17)		1.15 (0.84-1.60)		.40	
	**(12) Filling up spare time**												
			False	429 (55.42)		269 (59.65)		160 (49.54)		Ref		—	
			True	345 (44.57)		182 (40.35)		163 (50.46)		1.50 (1.12-2.00)		<.001	

^a^Not applicable.

^b^SQSU: sleep quality smartphone usage.

The analysis of various activities performed on smart devices during bedtime and their impact on the intention to stop using these devices in the next 3 months is presented in Table 2. The 3 most common activities were chatting via messaging platforms like WhatsApp, Snapchat, and Telegram, with 65.24% (505/774) of participants reporting this behavior. Next, 64.34% (498/774) of participants indicated they spent their time watching content on platforms such as YouTube, TikTok, and Instagram. Setting alarms, reminders, and tasks was also a frequent activity, with 60.07% (465/774) of participants engaging in this before bed. On the other hand, the least common activity performed on smart devices during bedtime was listening to music (100/774, 12.91%). Participants who used their devices for watching TV shows and movies (OR 1.70, 95% CI 1.27-2.29), listening to music (OR 1.68, 95% CI 1.10-2.57), and watching videos on platforms like YouTube, TikTok, and Instagram (OR 1.49, 95% CI 1.10-2.02) were less willing to stop using smart devices during bedtime in the next 3 months.

The analysis of different purposes for using smart devices during bedtime and their impact on the intention to stop using these devices in the next 3 months is presented in Table 2. The most common purposes were staying in touch with friends and family (432/774, 55.81%), entertainment (355/774, 45.86%), and filling up spare time (201/774, 44.57%). Participants who used their devices for entertainment (OR 1.94, 95% CI 1.45-2.59), finding information (OR 1.51, 95% CI 1.07-2.13), filling up spare time (OR 1.50, 95% CI 1.12-2.00), gaming (OR 1.54, 95% CI 1.08-2.20), and chatting via WhatsApp, Snapchat, Telegram, etc (OR 1.36, 95% CI 1.01-1.85) were less willing to stop using smart devices during bedtime in the next 3 months.

### Perceived Negative Effects of Smart Device Use on Sleep Quality and Next-Morning Well-Being

Over one-third of the participants (270/774, 35.23%) reported having poor sleep quality, as shown in Table 3. The OR analysis indicated that the perception of the negative influence of smartphone usage on sleep quality strongly impacted the intention to stop using these devices at bedtime. Participants who did not believe that using smart devices during bedtime negatively affected them the next morning were more likely to not intend to stop using their devices compared to those who did believe it (OR 3.53, 95% CI 2.53-4.91, *P*<.001). Similarly, participants who did not believe that smart device use during bedtime made them sleep less were 2.76 times more likely to not intend to stop (OR 2.76, 95% CI 1.97-3.86). Those who did not believe that using smart devices during bedtime caused a delayed sleep schedule were also more likely to not intend to stop compared to those who did believe it (OR 2.41, 95% CI 1.33-4.35).

The OR analysis also suggested that the perception of immediate negative health impacts influenced the intention to reduce or cease the use of smart devices at night. The most common issue was fatigue and drowsiness, with 62.01% (480/774) of participants experiencing these symptoms. Headaches were also prevalent, affecting 45.09% (349/774) of individuals, while 40.82% (316/774) reported mood swings and 40.69% (315/774) experienced a lack of focus and distraction. Participants who did not report any negative health observations had significantly higher odds (OR 5.25, 95% CI 2.36-11.69) of intending to stop using smart devices compared to those who did report negative health observations. Conversely, more than two-thirds of participants who experienced headaches, dry eyes, or lack of focus and distraction were less likely to intend to stop using smart devices at bedtime. Specifically, participants with headaches were 37% less likely (OR 0.63, 95% CI 0.47-0.85), those with dry eyes were 31% less likely (OR 0.69, 95% CI 0.51-0.94), and those with a lack of focus and distraction were 30% less likely (OR 0.70, 95% CI 0.52-0.95) to intend to stop compared to those without these symptoms.

**Table 3:** Perceived negative effects of smart device use on sleep quality and next-morning well-being among university students in Saudi Arabia.

Variables				Values (N=774)		Likely to stop using smart devices during bedtime in the next 3 months						
						Yes (n=451)		No (n=323)		Odds ratio (95% CI)		*P* value
**Using smart devices during bedtime makes you sleep less (SQPNE^a^_1), n (%)**												
	Yes		582 (75.19)		375 (83.15)		207 (64.09)		Ref		—^b^	
	No		192 (24.80)		76 (16.85)		116 (35.91)		2.76 (1.97-3.86)		<.001	
**Using smart devices during bedtime causes a delayed sleep schedule (SQPNE_2), n (%)**												
	Yes		724 (93.54)		432 (95.79)		292 (90.4)		Ref		—	
	No		50 (6.45)		19 (4.21)		31 (9.6)		2.41 (1.33-4.35)		<.001	
**Using smart devices during bedtime negatively affects oneself the next morning (SQPNE_3), n (%)**												
	Yes		561 (72.48)		374 (82.93)		187 (57.89)		Ref		—	
	No		213 (27.51)		77 (17.07)		136 (42.11)		3.53 (2.53-4.91)		<.001	
**After highly using the smart device the previous night, health observations the next morning (SQPNE_4)** **, n (%)**												
	**(1) Headache**											
		False	425 (54.90)		227 (50.33)		198 (61.3)		Ref		—	
		True	349 (45.09)		224 (49.67)		125 (38.7)		0.63 (0.47-0.85)		<.001	
	**(2) Dry eyes**											
		False	508 (65.63)		281 (62.31)		227 (70.28)		Ref		—	
		True	266 (34.36)		170 (37.69)		96 (29.72)		0.69 (0.51-0.94)		.02	
	**(3) Pain in the ear**											
		False	687 (88.75)		400 (88.69)		287 (88.85)		Ref		—	
		True	87 (11.24)		51 (11.31)		36 (11.15)		0.98 (0.62-1.54)		1.00	
	**(4) Fatigue and drowsiness**											
		False	294 (37.98)		160 (35.48)		134 (41.49)		Ref		—	
		True	480 (62.01)		291 (64.52)		189 (58.51)		0.77 (0.57-1.04)		.09	
	**(5) Lack of focus and distraction**											
		False	459 (59.30)		252 (55.88)		207 (64.09)		Ref		—	
		True	315 (40.69)		199 (44.12)		116 (35.91)		0.70 (0.52-0.95)		.02	
	**(6) Mood swings**											
		False	458 (59.17)		257 (56.98)		201 (62.23)		Ref		—	
		True	316 (40.82)		194 (43.02)		122 (37.77)		0.80 (0.60-1.07)		.15	
	**(7) Increased desire to have food rich in sugars and fats**											
		False	610 (78.81)		345 (76.50)		265 (82.04)		Ref		—	
		True	164 (21.18)		106 (23.50)		58 (17.96)		0.71 (0.49-1.01)		.07	
	**(8) Nothing**											
		False	738 (95.34)		443 (98.23)		295 (91.33)		Ref		—	
		True	36 (4.65)		8 (1.77)		28 (8.67)		5.25 (2.36-11.69)		<.001	
**Subjective sleep quality** **, n (%)**												
	**Sleep quality overall**											
		Very good	166 (21.44)		94 (20.84)		72 (22.29)		Ref		—	
		Fairly good	338 (43.66)		191 (42.35)		147 (45.51)		1.00 (0.69-1.46)		1.00	
		Fairly bad	200 (25.83)		118 (26.16)		82 (25.39)		0.90 (0.59-1.37)		0.67	
		Very bad	70 (9.04)		48 (10.64)		22 (6.81)		0.59 (0.33-1.08)		0.10	

^a^SQPNE: sleep quality perceived negative effects.

^b^Not applicable.

### Physical Sleep Disturbances Experienced During the Past Month

In terms of sleep latency, the majority of participants (209/774, 27%) reported having trouble falling asleep within 30 minutes 3 or more times per week, as shown in Table 4. Regarding sleep disturbances, waking up in the middle of the night or early morning occurred three or more times per week for 36.17% (280/774) of participants, making it a common problem. Use of sleep medications was not common, as 84.23% (652/774) of participants did not take sleep aids during the past month. Daytime dysfunction was significant for some participants, with 14.47% (112/774) struggling to stay awake 3 or more times per week during daily activities like driving or eating. Also, 31.39% (243/774) of participants reported that maintaining enthusiasm to complete tasks was somewhat of a problem, while 13.82% (107/774) found it to be a very big issue.

About 7.76% (35/451) of the participants who cough or snore loudly 3 or more times per week had significantly lower odds (OR 0.45, 95% CI 0.23-0.88, *P=*.02) of intending to stop using smart devices compared to those who did not. Similarly, 9.31% (42/451) of those who used sleep medications less than once per week had lower odds (OR 0.49, 95% CI 0.27-0.89, *P=*.01) of intending to stop using smart devices compared to those who did not use sleep medications. Over a quarter of participants who needed to get up to use the bathroom less than once per week (119/451, 26.39%) or once or twice per week (91/451, 20.18%) had lower odds of intending to stop using smart devices at bedtime compared to those who did not need to get up (OR 0.63, 95% CI 0.43-0.92, *P*=.01 for less than once per week; OR 0.63, 95% CI 0.42-0.96, *P*=.03 for once or twice per week). About 21.51% (97/451) of the participants who could not breathe comfortably less than once per week had lower odds (OR 0.67, 95% CI 0.46-0.99, *P=*.05) of intending to stop using smart devices compared to those who did not have breathing issues. Finally, 26.61% (120/451) of the participants who felt too cold once or twice per week had lower odds (OR 0.62, 95% CI 0.42-0.91, *P=*.01) of intending to stop using smart devices compared to those who did not feel too cold.

**Table 4:** Physical sleep disturbances experienced during the past month by university students in Saudi Arabia.

PSQI^a^ components				Values (N=774)		Likely to stop using smart devices during bedtime in the next 3 months						
						Yes (n=451)		No (n=323)		Odds ratio (95% CI)		*P* value
**Sleep latency, n (%)**												
	**Cannot get to sleep within 30 minutes**											
		Not during the past month	201 (25.96)		120 (26.61)		81 (25.08)		Ref		—^b^	
		Less than once per week	173 (22.35)		100 (22.17)		73 (22.60)		1.08 (0.71-1.63)		.75	
		Once or twice per week	191 (24.67)		110 (24.39)		81 (25.08)		1.09 (0.72-1.63)		.68	
		Three or more times per week	209 (27.00)		121 (26.83)		88 (27.24)		1.07 (0.72-1.59)		.76	
**Sleep disturbance, n (%)**												
	**Wake up in the middle of the night or early morning**											
		Not during the past month	187 (24.16)		101 (22.39)		86 (26.63)		Ref		—	
		Less than once per week	125 (16.14)		79 (17.52)		46 (14.24)		0.68 (0.43-1.08)		.12	
		Once or twice per week	182 (23.51)		102 (22.62)		80 (24.77)		0.92 (0.61-1.38)		.75	
		Three or more times per week	280 (36.17)		169 (37.47)		111 (34.37)		0.77 (0.53-1.12)		.18	
	**Have to get up use to the bathroom (SQDPM^c^_1)**											
		Not during the past month	287 (37.08)		150 (33.26)		137 (42.41)		Ref		—	
		Less than once per week	188 (24.28)		119 (26.39)		69 (21.36)		0.63 (0.43-0.92)		.01	
		Once or twice per week	144 (18.60)		91 (20.18)		53 (16.41)		0.63 (0.42-0.96)		.03	
		Three or more times per week	155 (20.02)		91 (20.18)		64 (19.81)		0.77 (0.51-1.14)		.22	
	**Cannot breathe comfortably (SQDPM_2)**											
		Not during the past month	453 (58.52)		251 (55.65)		202 (62.54)		Ref		—	
		Less than once per week	150 (19.37)		97 (21.51)		53 (16.41)		0.67 (0.46-0.99)		.05	
		Once or twice per week	94 (12.14)		56 (12.42)		38 (11.76)		0.84 (0.53-1.32)		.49	
		Three or more times per week	77 (9.94)		47 (10.42)		30 (9.29)		0.79 (0.48-1.29)		.38	
	**Cough or snore loudly (SQDPM_3)**											
		Not during the past month	580 (74.93)		330 (73.17)		250 (77.40)		Ref		—	
		Less than once per week	88 (11.36)		51 (11.31)		37 (11.46)		0.95 (0.60-1.50)		.90	
		Once or twice per week	59 (7.62)		35 (7.76)		24 (7.43)		0.90 (0.52-1.56)		.78	
		Three or more times per week	47 (6.07)		35 (7.76)		12 (3.72)		0.45 (0.23-0.88)		.02	
	**Feel too cold (SQDPM_4)**											
		Not during the past month	262 (33.85)		138 (30.60)		124 (38.39)		Ref		—	
		Less than once per week	144 (18.60)		88 (19.51)		56 (17.34)		0.70 (0.46-1.07)		.11	
		Once or twice per week	187 (24.16)		120 (26.61)		67 (20.74)		0.62 (0.42-0.91)		.01	
		Three or more times per week	181 (23.38)		105 (23.28)		76 (23.53)		0.80 (0.54-1.18)		.28	
	**Feel too hot**											
		Not during the past month	317 (40.95)		179 (39.69)		138 (42.72)		Ref		—	
		Less than once per week	175 (22.60)		104 (23.06)		71 (21.98)		0.88 (0.60-1.28)		.56	
		Once or twice per week	165 (21.31)		97 (21.51)		68 (21.05)		0.90 (0.62-1.33)		.69	
		Three or more times per week	117 (15.11)		71 (15.74)		46 (14.24)		0.84 (0.54-1.29)		.44	
	**Have bad dreams**											
		Not during the past month	271 (35.01)		155 (34.37)		116 (35.91)		Ref		—	
		Less than once per week	241 (31.13)		139 (30.82)		102 (31.58)		0.98 (0.69-1.39)		.92	
		Once or twice per week	170 (21.96)		98 (21.73)		72 (22.29)		0.98 (0.66-1.44)		>.99	
		Three or more times per week	92 (11.88)		59 (13.08)		33 (10.22)		0.74 (0.45-1.21)		.27	
	**Have pain**											
		Not during the past month	381 (49.22)		213 (47.23)		168 (52.01)		Ref		—	
		Less than once per week	156 (20.15)		90 (19.96)		66 (20.43)		0.92 (0.63-1.35)		.77	
		Once or twice per week	125 (16.14)		78 (17.29)		47 (14.55)		0.76 (0.50-1.15)		.21	
		Three or more times per week	112 (14.47)		70 (15.52)		42 (13.00)		0.76 (0.49-1.17)		.23	
**Use of sleep medications, n (%)**												
	**Taken medicine to help you sleep (prescribed or “over the counter”) (SQDPM_5)**											
		Not during the past month	652 (84.23)		367 (81.37)		285 (88.24)		Ref		—	
		Less than once per week	58 (7.49)		42 (9.31)		16 (4.95)		0.49 (0.27-0.89)		.01	
		Once or twice per week	37 (4.78)		26 (5.76)		11 (3.41)		0.54 (0.26-1.12)		.12	
		Three or more times per week	27 (3.48)		16 (3.55)		11 (3.42)		0.88 (0.40-1.93)		.84	
**Daytime dysfunction, n (%)**												
	**Having trouble staying awake while driving, eating meals, or engaging in social activity**											
		Not during the past month	369 (47.67)		206 (45.68)		163 (50.46)		Ref		—	
		Less than once per week	161 (20.80)		103 (22.84)		58 (17.96)		0.71 (0.48-1.04)		.08	
		Once or twice per week	132 (17.05)		69 (15.30)		63 (19.50)		1.15 (0.77-1.71)		.54	
		Three or more times per week	112 (14.47)		73 (16.19)		39 (12.07)		0.67 (0.43-1.04)		.08	
	**Keeping up enough enthusiasm to get things done**											
		No problem at all	202 (26.09)		112 (24.83)		90 (27.86)		Ref		—	
		Only a very slight problem	222 (28.68)		137 (30.38)		85 (26.32)		0.77 (0.52-1.13)		.20	
		Somewhat of a problem	243 (31.39)		137 (30.38)		106 (32.82)		0.96 (0.66-1.40)		.84	
		A very big problem	107 (13.82)		65 (14.41)		42 (13.00)		0.80 (0.49-1.29)		.39	

^a^PSQI: Pittsburgh Sleep Quality Index.

^b^Not applicable.

^c^SQDPM: sleep quality during the past month.

### Predictors of the Intention to Stop Using Smart Devices at Bedtime

Path analysis was conducted to understand the complex relationships between the measured variable intended to stop using smart device usage and the latent variables, which were SQSU (presented in Table 2), SQPNE (presented in Table 3), and SQDPM (presented in Table 4). All the demographic factors in Table 1 were removed from the analysis as they did not approach the significance threshold of <.05. To improve the model fit, only the significant variables (<.05) from Tables 2-4 were included. SEM plot indicated that the model had an excellent fit with a nonsignificant chi-square, a CFI of 0.845, and a RMSEA value of 0.039 (95% CI 0.032-0.046).

All the factors except SQSU_7 (purposes=–0.09, *P*>.05) had significant factor loadings (path coefficients) with latent variables SQSU, SQPNE, and SQDPM. People using smart devices every night (SQSU_1; 0.36, *P*<.05), 3 hours or more time spent on smart devices at bedtime (SQSU_2; –0.35, *P*<.05), using the smart device frequently after lights were turned off (SQSU_3; 0.49, *P*<.05), using headphones frequently during bedtime (SQSU_4; 0.39, *P*<.05), awakened frequently by the smart device at night (SQSU_5; 0.25, *P*<.05), activities (SQSU_6; –0.18, *P*<.05) had significant path coefficients with latent variable SQSU.

People who perceived using smart devices during bedtime led to sleeplessness (SQPNE_1; 0.58, *P*<.05), caused delayed sleep schedule (SQPNE_2; 0.29, *P*<.05), negatively affected oneself the next morning (SQPNE_3; 0.71, *P*<.05), and affected their health (SQPNE_4; 0.24, *P*<.05) had significant path coefficients with latent variable SQPNE.

People woke up in the middle of the night or early morning, had to get up to use the bathroom (SQDPM_1; 0.35, *P*<.05), could not breathe comfortably (SQDPM_2; 0.64, *P*<.05), coughed or snored loudly (SQDPM_3; 0.41, *P*<.05), felt cold (SQDPM_4; 0.42, *P*<.05), and took medicine to help sleep (SQDPM_5; 0.33, *P*<.05) had significant path coefficients with latent variable SQDPM.

**Figure 1:**
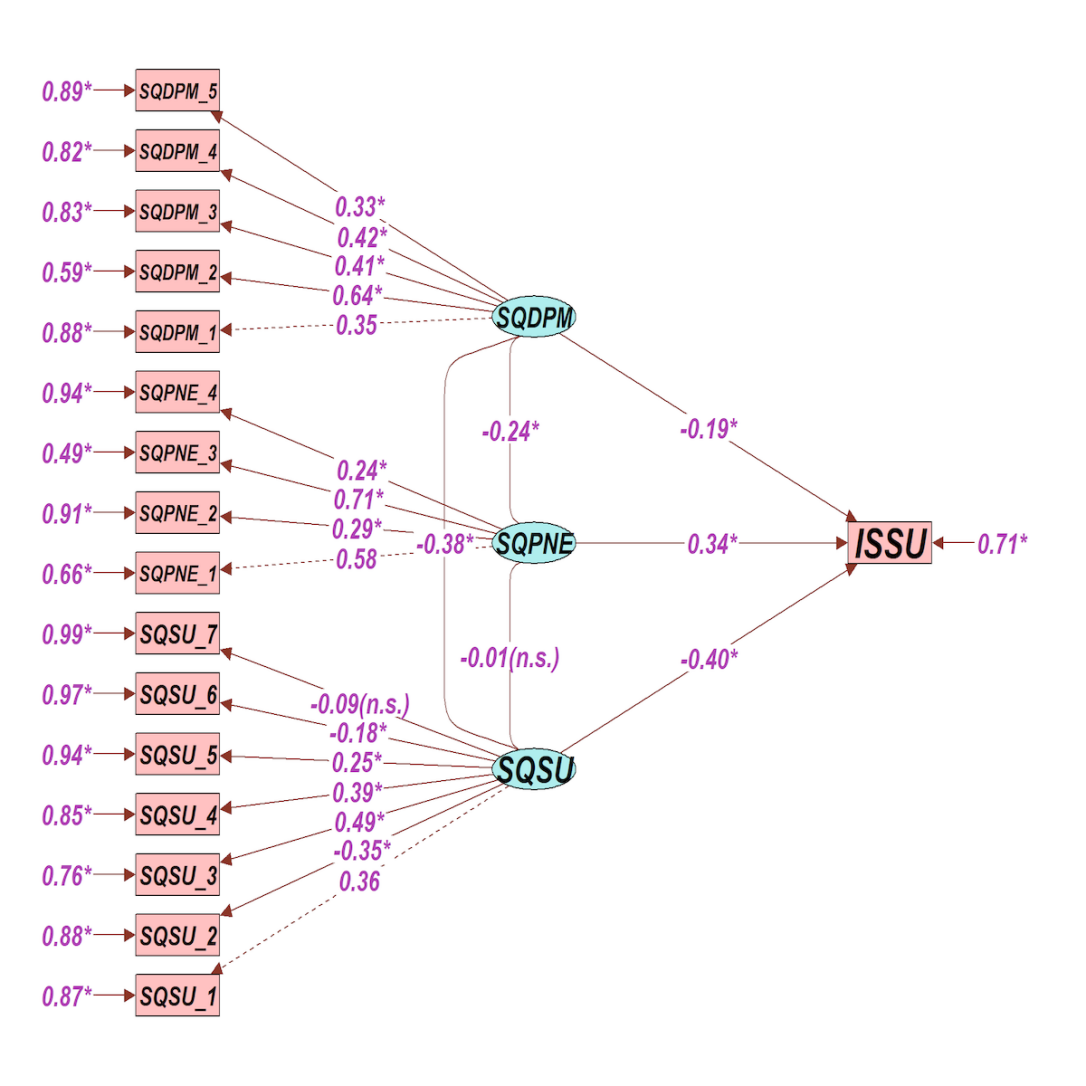
Path analysis of predictors influencing university students’ intention to stop using smart devices at bedtime in Saudi Arabia. An asterisk indicates a 2-tailed *P* value <.05. ISSU: intended to stop using smart device usage; SQDPM: sleep quality during the past month; SQPNE: sleep quality perceived negative effects; SQSU: sleep quality smartphone usage.

The 3 obtained factors have path coefficients with ISSU (intended to stop using smart device usage). SQSU=–0.40, SQPNE=0.34, and SQDPM=–0.19 were significant and there was no evidence of collinearity in the model as shown in Figure 1. All items of the model had significant paths as well as correlations with respective factor domains. Factor structures remained intact, and all paths were significant.

## Discussion

### Principal Findings

This study investigated the factors that influence university students’ intention to stop using smart devices at bedtime in Saudi Arabia. It focused on behavioral habits, perceived negative effects on sleep quality, and physical sleep disturbances. The findings provide insights into how smartphone usage behaviors and sleep-related perceptions interplay, influencing students’ intentions to modify their presleep device habits, as follows.

### Behavioral Factors and Smart Device Usage Habits

The results from the analysis of smartphone usage habits (Table 2) revealed that frequent nightly use and the use of smart devices after lights are turned off were significantly associated with the intention to stop using these devices at bedtime. Specifically, using smart devices after lights out (path coefficient=0.49) was the strongest behavioral predictor, indicating that students who engage in late-night device use may recognize its disruptive effects on sleep and are thus more inclined to reduce or stop this behavior. This aligns with previous studies that highlight the adverse effects of presleep exposure to light from screens, which delays sleep onset [13,17].

However, spending more than 3 hours on devices (path coefficient=–0.35) and engaging in multiple activities (path coefficient=–0.18) were negatively associated with the intention to stop using smart devices at bedtime. This suggests that heavy users may be more resistant to behavioral change, possibly due to their addiction to using smart devices [1]. This aligns with previous studies that have identified higher levels of smartphone addiction as barriers to changing screen time habits [27,28].

Moreover, engaging in more activities on the device also decreased the likelihood of stopping using smart devices (path coefficient=–0.18), which may indicate that participants perceive these activities (eg, watching videos and chatting) as integral parts of their nightly routine [29,30]. These results are consistent with previous studies that link high-frequency smart device use to a reluctance to disengage from these devices, possibly due to increased dependency on smart devices for both academic and social purposes [27,31].

### Perceived Negative Effects on Sleep Quality and Well-Being

The most significant predictors of the intention to stop using smart devices were related to perceived negative effects on sleep quality and next-morning well-being (Table 3). The strongest predictor was the belief that smart device usage negatively affects next-morning well-being (path coefficient=0.71). This suggests that students who experience morning fatigue, mood disturbances, or difficulty focusing are more likely to stop using their devices before sleep. This is consistent with previous studies that demonstrate how poor sleep quality directly influences daytime functioning [19], especially in academic contexts where cognitive performance is critical [15,30,31].

Similarly, the perception of sleeplessness due to smart device use was also a strong predictor (path coefficient=0.58). This indicates that university students who struggle to sleep because of their devices are more likely to intend to stop using them. This is consistent with previous studies on the disruptive effects of presleep screen time, which has been linked to increased sleep latency and poorer overall sleep quality [13,32]. In addition, the perception of delayed sleep schedules (path coefficient=0.29) also contributed to the intention to stop, further reinforcing the idea that students recognize the role of smart devices in disrupting their sleep timing.

### Physical Sleep Disturbances and Health Implications

The results from physical sleep disturbances (Table 4) highlighted how certain health issues experienced during the night are significantly linked with the intention to stop using smart devices. Difficulty breathing comfortably during sleep (path coefficient=0.64) was the most prominent physical predictor, suggesting that students who experience physical discomforts, such as respiratory issues or muscle tension, may attribute these problems to their use of smart devices at bedtime and are therefore more likely to reduce their usage. This is consistent with studies that have found links between prolonged device use in bed and poor posture or breathing discomfort, potentially due to prolonged screen time in awkward positions [33].

Other sleep disturbances, such as waking up during the night, snoring or coughing, and feeling too cold, were also positively associated with the intention to stop using smart devices at bedtime. These findings indicate that students who experience sleep disruptions are more likely to modify their behavior to improve their sleep quality [8,20]. Furthermore, students who reported taking sleep medications were more likely to intend to stop using smart devices, suggesting that those with more severe sleep problems may be more motivated to address contributing factors like bedtime device use [31].

### Implications for University Students and Public Health Interventions

These findings have significant implications for interventions targeting sleep hygiene among university students, particularly in Saudi Arabia. Given that the strongest predictors of the intention to stop using smart devices were perceived negative effects on next-morning well-being and physical sleep disturbances, public health initiatives should focus on raising awareness about these health risks [34]. Particularly, educational campaigns should emphasize how devices used before bed negatively impact not only sleep quality but also cognitive functioning, mood, and overall well-being the next day [35].

Interventions should also incorporate behavior change theories, such as the Transtheoretical Model and the Theory of Planned Behavior [36,37], to understand and address resistance to change. Many heavy device users, despite recognizing negative outcomes, may rely on their devices for emotional regulation or social engagement [5,16], making these activities a barrier to change. Tailored strategies should therefore target these specific behaviors, offering alternative coping mechanisms like mindfulness exercises, relaxation techniques [38], or structured bedtime routines to gradually reduce dependency on devices [39]. Furthermore, strategies that encourage limiting screen time after lights out and reducing device-related disruptions, such as turning off notifications or using blue light filters, could be effective in promoting healthier sleep habits [32,34].

### Strengths, Limitations, and Future Directions

This study has several strengths. First, its multidimensional approach examined not only behavioral habits but also perceived negative effects and physical sleep disturbances, offering a holistic view of the factors that influence the intention to stop using smart devices. Second, the use of a validated scale such as PSQI adds rigor to the assessment of sleep quality. Finally, the application of path analysis and SEM allowed for an in-depth analysis of the relationships between latent variables, supporting the interpretation of complex patterns.

However, there are some limitations. The cross-sectional design limits the ability to establish causality between smart device usage and sleep disturbances [40].

The reliance on self-reported data introduces potential biases, including recall bias (where participants may not accurately remember behaviors or experiences) and social desirability bias (where participants may provide what they perceive to be more acceptable responses). These biases could influence the accuracy of the findings [41]. Furthermore, the open recruitment approach via social media platforms prevented determining an exact participation rate [42]. Although a large sample (n=774) of university students from Saudi Arabia may improve the applicability of the findings to this population, replication in other cultural contexts is necessary to assess broader generalizability and refine intervention strategies.

Future studies should consider longitudinal research to establish causality and interventional studies to test specific strategies for reducing device use at bedtime [16,26]. Also, investigating different types of smart device use (eg, browsing social media, gaming, chatting, and studying) would provide more nuanced insights. In addition, future studies should investigate the interactions between smart device use, sleep hygiene, and mental health such as anxiety, depression, or stress levels because understanding these interactions could inform more tailored interventions for improving sleep among university students [5].

### Conclusions

This study provides valuable insights into the factors influencing university students’ intention to stop using smart devices at bedtime, particularly in the Saudi Arabian context. While behavioral habits, such as frequent device use and activities, are important, the most significant predictors of behavior change are perceived negative effects on well-being and physical sleep disturbances. These findings highlight the importance of addressing both the psychological and physiological impacts of smart device use in interventions aimed at improving sleep quality among university students. Future studies should explore how these interventions can be effectively implemented and sustained over time to mitigate the negative effects of excessive bedtime device use.

## Abbreviations

CFI: comparative fit index
OR: odds ratio
PSQI: Pittsburgh Sleep Quality Index
RMSEA: root mean square error of approximation
SEM: sequencing equation modeling
STROBE: Strengthening the Reporting of Observational Studies in Epidemiology
SQDPM: sleep quality during the past month
SQPNE: sleep quality perceived negative effects
SQSU: sleep quality smartphone usage

## Data Availability

The datasets generated during and/or analyzed during this study are available from the author on reasonable request.
